# Effect of the Flame Retardants and Glass Fiber on the Polyamide 66/Polyphenylene Oxide Composites

**DOI:** 10.3390/ma15030813

**Published:** 2022-01-21

**Authors:** Zhenya Zhang, Mingcheng Yang, Kunpeng Cai, Yang Chen, Shubo Liu, Wentao Liu, Jilin Liu

**Affiliations:** 1 Institute of Isotope, Henan Academy of Sciences Co., Ltd., No. 7 Songshan South Road, Zhengzhou 450015, China; zhangzhenya0924@126.com (Z.Z.); chenyang.1992@outlook.com (Y.C.); liushubo1990@126.com (S.L.); 2 Department of Extreme Environmental Coatings, Korea Institute of Materials Science, Changwon 797, Korea; 3 Jiaozuo Tongfu Technology Co., Ltd., Jiaozuo 454100, China; 4 School of Materials Science and Engineering, Zhengzhou University, No. 100 Science Avenue, Zhengzhou 450001, China; ckp9557@126.com (K.C.); wtliu@zzu.edu.cn (W.L.); 13134465689@163.com (J.L.)

**Keywords:** PA66/PPO composites, limiting oxygen index, UL-94 vertical burning level, mechanical properties, thermal decomposition

## Abstract

In this work, polyamide 66/polyphenylene oxide (PA66/PPO) composites, including the flame retardants 98 wt% aluminum diethylphosphinate + 2 wt% polydimethylsiloxane (P@Si), Al(OH)_3_-coated red phosphorus (RP*), and glass fiber (GF), were systematically studied, respectively. The limiting oxygen index (LOI), UL-94 vertical burning level, and thermal and mechanical properties of the PA66/PPO composites were characterized. The results showed that the P@Si and RP flame retardants both improved the LOI value and UL-94 vertical burning level of the PA66/PPO composites, and PA66/PPO composites passed to the UL-94 V-0 level when the contents of P@Si and RP* flame retardants were 16 wt% and 8 wt%. On the other hand, the mechanical properties of the PA66/PPO composites were reduced from a ductile to a brittle fracture mode. The addition of GF effectively made up for these defects and improved the mechanical properties of the PA66/PPO composites containing the P@Si and RP*, but it did not change the fracture mode. P@Si and RP* flame retardants improved the thermal decomposition of PA66/PPO/GF composites and reduced the maximum mass loss rates, showing that the PA66/PPO/GF composites containing the P@Si and RP* flame retardants could be used in higher-temperature fields.

## 1. Introduction

Polyamide (PA) is a thermoplastic engineering plastic, possessing mechanical properties, molding properties, chemical resistance, and electrical insulation [1,2,3,4]. PA66 is a typical polyamide, containing many amide bonds in the main chain, possessing excellent processing properties and lower melt viscosity [5]. However, the limiting oxygen index (LOI) value of pure PA66 is approximately 22.5, indicating that it possesses poor flame retardancy [6]. In addition, the burning and smoke-releasing problems of PA66 cause harm to people and the environment, limiting its extensive application in high-temperature fields [7,8]. Therefore, PA66 needs to be alloyed to improve its properties [9]. Polyphenylene oxide (PPO) is an amorphous polymer which possesses excellent thermal stability, mechanical strength, and flame-retardant properties due to its unique molecular structure and its LOI value of approximately 28 [10,11,12,13]. Therefore, PA66/PPO composites with compatibilizers have been extensively studied.

Son et al. fabricated PA66/PPO/elastomer using a one-step method. The physical properties of the composite were similar to those of PA66/PPO fabricated using two steps [14]. Furthermore, their method also reduced the production cost. Zhang et al. studied the preparation of PA66/PPO using the boric acid ester as a compatibilizer; the compatibilization between PA66 and PPO was remarkably improved [15]. Li et al. studied the rheological properties of PA66/PPO-g-MAH blends, which showed a higher viscosity because the chemical reaction occurred in PA66/PPO-g-MAH blends during melt blending, increasing the molecular weight and branch number [16]. In addition, the mechanical properties of PA66/PPO-g-MAH blends were also improved compared to PA66/PPO blends. Zhang et al. reported that the HIPS-g-MAH compatibilizer of 7 wt% improved the mechanical properties of PA66/PPO [17]. The above studies have effectively solved the problem of interface compatibility between PA66 and PPO. In addition, improvements in the mechanical properties of PA/PPO blends have been studied, such as the use of PA46/PPO blends. Ran et al. studied the use of polyurethane-coated carbon fiber to strengthen the mechanical properties of PA46/PPO blends. The results showed that the carbon fiber remarkably enhanced tensile strength and reduced volumetric wear [12]. However, the improvement of the mechanical properties of PA66/PPO using glass fiber or other additives has been rarely reported.

The flame-retardant performance of polymers has been studied, for example, in studies of PA6 [18,19,20] and PA66 [21]. Kanno et al. reported that additive-type and reactive-type organic phosphorus flame retardants both improved the flame-retardant performance of PA66 and enable it to reach the UL-94 V-0 level [21]. Furthermore, halogen-free flame retardants have been used to improve the property of flame-retardancy because they are environment-friendly [22,23,24,25,26,27]. Although the PA66/PPO composite combines the advantages of PA66 and PPO, it has poor flame retardancy resistance and this issue has not been resolved. In this study, commercial halogen-free flame retardants, Al(OH)_3_-coated red phosphorus and aluminum diethylphosphinate (ADP) mixed with polydimethylsiloxane, were selected as flame retardants to add into the PA66/PPO composite. Then, glass fibers (GF) were added to the PA66/PPO composites to improve their mechanical properties. Finally, the thermal and mechanical properties of the PA66/PPO composites were measured.

## 2. Experimental Methods

### 2.1. Materials

PA66 and PPO particles were chosen as the raw materials. The HIPS-g-MAH compatibilizer prepared in the laboratory was used to improve the compatibility in PA66/PPO composites; for more specific preparation details, refer to [17]. Commercial flame retardants 98 wt% aluminum diethylphosphinate + 2 wt% polydimethylsiloxane (abbreviation: P@Si) (particle size < 10 μm)) and Al(OH)_3_-coated red phosphorus (abbreviation: RP*) (particle size < 10 μm) were used in this work. Non-alkali non-twisted glass fiber (GF), with excellent fatigue resistance, mechanical properties, and high-temperature resistance, was used to improve the mechanical properties, supported by the Jushi Group Co., Ltd., Jiaxing, China. Detailed information on all materials is shown in Table 1.

### 2.2. Preparation of PA66/PPO Composite

Two kinds of halogen-free flame-retardant PA66/PPO composites (PA66/PPO-P@Si and PA66/PPPO-RP*), including an HIPS-g-MAH compatibilizer of 7 wt%, were prepared using the extrusion and injection molding. Prior to extrusion, the PA66 and PPO particles were dried in a vacuum oven at 110 °C for 4 h to remove the moisture. PA66, PPO, the HIPS-g-MAH compatibilizer, flame-retardants, and GF were uniformly mixed in different proportions. Next, PA66/PPO composite particles were prepared using a twin-screw extruder (SHJ-20, Nanjing Giant Machinery Co., Ltd., Nanjing, China). The barrel temperature ranged from 250 °C to 270 °C, and the die temperature was 265 °C ± 2 °C. In addition, the screw speed was 160 rpm. Then PA66/PPO samples were manufactured using an injection molding machine (PL860/260, Wuxi Haitian Machinery Co., Ltd., Wuhan, China) with a mold area temperature of 65 °C–75 °C. The fixed weight ratio of PA66 and PPO in PA66/PPO composites was 7.5/2.0.

### 2.3. Characterization of PA66/PPO Composites

The tensile and bend strength of the dumbbell PA66/PPO composites were conducted on a Microcomputer control electronic universal testing machine (RGM-3010, Shenzhen Rigel Instrument Co., Ltd., Shenzhen, China) with a spline size of 80 mm × 10 mm × 4 mm. The notched impact strength of the PA66/PPO composites was tested using an Izod impact tester (XJU-5.5, Chengde, China), and the notch shape was a V-shaped mouth of 45°. The LOI values of the PA66/PPO composites with 80 mm × 10 mm × 4 mm were tested using a digital oxygen index meter (JF-3, Nanjing Jionglei Instrument Equipment Co., Ltd., Nanjing, China). In addition, the UL-94 vertical burning level of PA66/PPO composites of 130 mm × 13 mm × 3 mm was tested using a horizontal-vertical flammability tester (5402, Suzhou Yangyi Vouch Testing Technology Co., Ltd., Suzhou, China). The final results presented above are the averages of five test values each. The fracture surface morphologies of PA66/PPO composites were characterized by means of scanning electron microscopy (SEM, S-4800, Hitachi, Japan). Differential scanning calorimetry (DSC) curves were tested using Netzsch DSC 204 (Selb-Plößberg, Germany) equipment, and the TGA and DGA curves were obtained using Netzsch STA-409 (Selb-Plößberg, Germany) analysis under an N_2_ atmosphere, with a heating rate of 10 °C/min. Heat distortion temperature (HDT) analysis of PA66-PPO compositions was performed using a Thermal deformation Vicat Softening Temperature Tester (HDT/V1002, Chengde, China); the heating rate was 2 °C/min and the load was 1.80 MPa.

## 3. Results

### 3.1. Flame Retardant Performance of the PA66/PPO Composites

**Figure 1:**
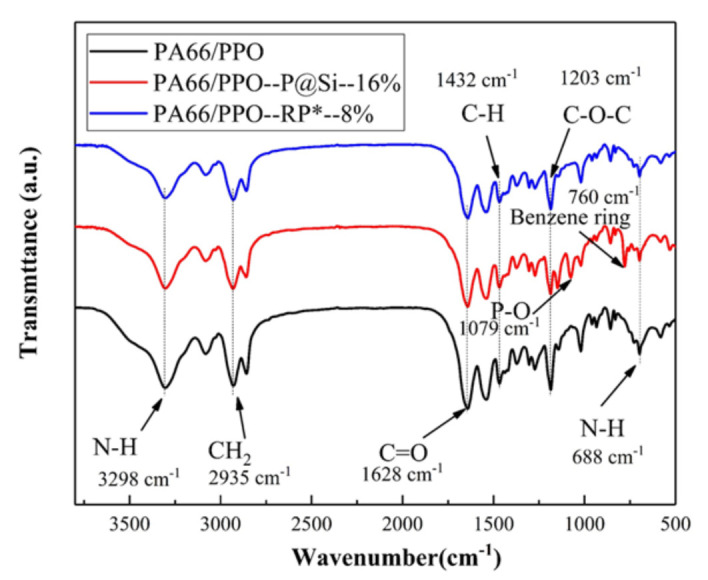
FT-IR spectra of PA66/PPO composites.

Figure 1 shows the FT-IR spectra of PA66/PPO composites. The typical absorption peaks of PA66 were found at 3298 cm^−1^ (N-H stretching), 2935 cm^−1^ (CH_2_ stretching), 1628 cm^−1^ (C = O stretching, amide I), and 688 cm^−1^ (N-H bending vibration) [28,29] in three samples. On the other hand, 1432 cm^−1^ (C-H stretching) and 1203 cm^−1^ (C-O-C stretching) [30,31] were the typical absorption peaks of PPO. PA66 and PPO possessed good compatibility under the action of the HIPS-g-MAH compatibilizer [17]. In addition, the stretching vibration absorption peaks of P-O and the benzene ring of aluminum diethylphosphinate (ADP) appeared at 1079 cm^−1^ and 760 cm^−1^ [32] in the PA66/PPO–P@Si–16% composites, meaning that flame-retardant P@Si was detected. However, the typical absorption peaks of RP* were not found in the PA66/PPO–RP*–8% composites. It is possible that the red phosphorus was wholly coated by Al(OH)_3_. No chemical reactions occurred because the P@Si and RP* flame retardants are of the additive type rather than the reactive type.

**Figure 2:**
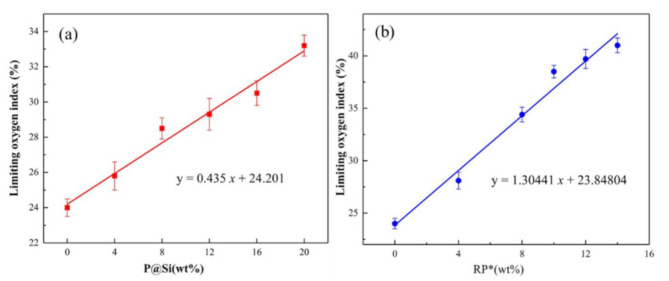
Limiting oxygen index of the (**a**) PA66/PPO–P@Si and (**b**) PA66/PPO–RP* composites.

The LOI measurement and vertical burning test (UL-94) are the two basic flammability evaluation methods that investigate the effect of flame retardants on improving the flame retardancy of composites. The LOI values and UL-94 levels of PA66/PPO composites are shown in Figure 2 and listed in Table 2. P@Si and RP* flame retardants were able to effectively inhibit the combustion of PA66/PPO composites. The PA66/PPO composites passed the UL-94 V-0 level when the contents of P@Si and RP* flame retardants were 16 wt% and 8 wt%, respectively. Increasing the content of the P@Si and RP* flame retardants, the PA66/PPO compositions remained at the UL-94 V-0 level. In Figure 2a, the relationship between the LOI values of PA66/PPO composites and the content of P@Si was linear, which conformed to the equation y = 0.435 x + 24.201. The LOI value of the PA66/PPO composite showed a linear increase with an increase in the content of the RP* flame retardant, which was in accordance with the equation y = 1.30441 x + 23.84804, as shown in Figure 2b. According to these equations, PA66/PPO composites containing the RP* flame retardant had a better flame-retardant effect.

### 3.2. Mechanical Properties of PA66/PPO Composites

**Figure 3:**
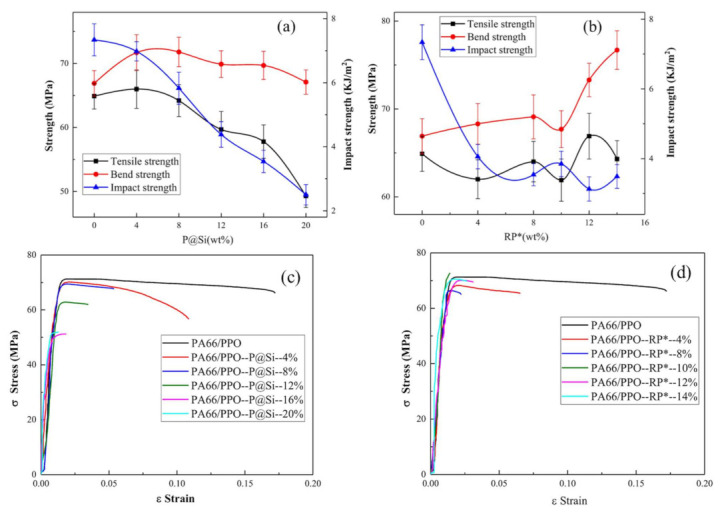
Mechanical properties of (**a**) PA66/PPO–P@Si and (**b**) PA66/PPO–RP* composites, and stress-strain curves of (**c**) PA66/PPO–P@Si and (**d**) PA66/PPO–RP* composites.

The mechanical properties of PA66/PPO composites with different flame retardant ratios are shown in Figure 3. The bend strength of the PA66/PPO–P@Si increased first and then slightly decreased with the increasing P@Si flame retardant weight ratios, as shown in Figure 3a. However, the tensile and impact strength decreased with an increase in the P@Si content. The tensile, bend, and impact strength of PA66/PPO–P@Si–16% were 57.8 ± 2.6 MPa, 69.7 ± 2.2 MPa, and 3.54 ± 0.35 KJ/m^2^, respectively. The tensile strength and impact strength were reduced by 13.6% and 51.8%, respectively, implying that the P@Si flame retardant reduced the mechanical properties of PA66/PPO composites. As shown in Figure 3b, the tensile strength of PA66/PPO–RP* fluctuated around 65 Mpa, without drastic changes. However, the bend strength displayed an increasing trend with increase in the RP* content, and the impact strength rapidly decreased and then remained stable. For example, when the RP* contents were 8 wt% and 14 wt%, the tensile strengths were 64.0 ± 2.30 MPa and 64.3 ± 2.13 MPa. In comparison, the corresponding impact strengths decreased by 51.8% and 52.5%, respectively. Figure 3c,d show the stress-strain curves of the PA66/PPO–P@Si and PA66/PPO–RP* composites following the uni-axial tensile test at room temperature. For the PA66/PPO composite, the strain ε of 0.18 was higher than that of the PA66/PPO–P@Si and PA66/PPO–RP* composites. The strain ε decreased with an increase in the flame retardant contents in the PA66/PPO–P@Si and PA66/PPO–RP* composites, which meant that the ductility behavior of PA66/PPO composites decreased. The PA66/PPO–P@Si–20% and PA66/PPO–RP*–14% composites displayed a brittle fracture mode. Consequently, P@Si and RP* flame retardants reduced the mechanical properties of PA66/PPO composites and changed the fracture mode.

**Figure 4:**
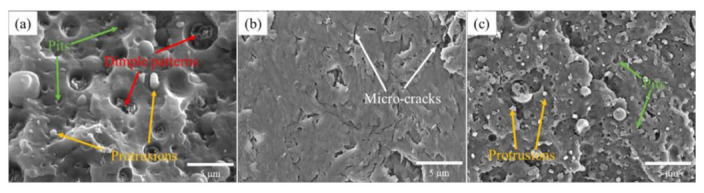
SEM images of the notch impact surface of the PA66/PPO composites; (**a**) PA66/PPO, (**b**) PA66/PPO–P@Si–16%, and (**c**) PA66/PPO–RP*–8%.

Figure 4 shows the SEM images of the notch impact surfaces of different samples. Many protrusions (orange arrows), pits (green arrows), and dimple patterns (red arrows) were found on the surface of the PA66/PPO compositions, indicating that the impact fracture was a ductile fracture, as shown in Figure 4a. However, in Figure 4b, the impact fracture morphologies of PA66/PPO–P@Si–16% showed a relatively smooth surface and some micro-cracks (white arrows) were found in the SEM image, showing a brittle fracture mode, rather than the ductile fracture mode. The results corresponded to the reduction of impact strength, as shown in Figure 3a. Furthermore, although some smaller protrusions and pits were found on the surface in Figure 4c, the dimple patterns disappeared on the surface, indicating that the impact fracture mode of the PA66/PPO–RP*–8% composite was not the ductile fracture mode. As a result, the impact strength of PA66/PPO–RP* composites decreased, as shown in Figure 3b.

**Figure 5:**
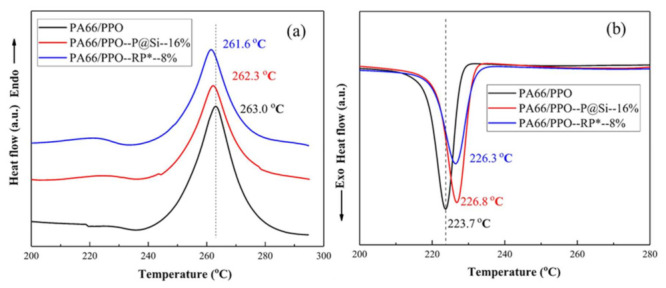
DSC patterns of the PA66/PPO composites: (**a**) heating process and (**b**) cooling process.

Figure 5 shows the DSC curves of the PA66/PPO composites. Endo and Exo stand for endothermic and exothermic heat. In Figure 5a, the melting peaks of PA66/PPO composites PA66/PPO–P@Si–16% and PA66/PPO–RP*–8% appeared at 263.0 °C, 262.3 °C, and 261.6 °C, respectively. The addition of flame retardant did not increase the melting temperature of PA66/PPO compositions but slightly lowered the melting temperature of PA66/PPO composites. The reduction of the melting temperature of PA66/PPO–P@Si–16% and PA66/PPO–RP*–8% composites was attributed to the decrease in the crystallinity of the composite material under the action of the flame retardant. Figure 5b shows the DSC curves of PA66/PPO composites during the cooling process. The cold crystallization peaks of the PA66/PPO PA66/PPO–P@Si and PA66/PPO–RP* composites were observed at 223.7 °C, 226.8 °C, and 226.3 °C, respectively. The addition of flame retardant increased the temperature of the cold crystallization peak. The cold crystallization peak of the PA66/PPO composites was relatively sharp, meaning that the crystal formation and growth rate of the PA66/PPO composites was fast after reaching a sufficient degree of subcooling. The introduction of flame retardant limited the migration and rearrangement of the molecular chains of PA66/PPO composites, reduced the rate of crystal formation and growth, causing the grain size distribution to be uneven and meaning that the degree of crystallinity was not perfect.

### 3.3. Mechanical Properties of PA66/PPO/GF Composites

**Figure 6:**
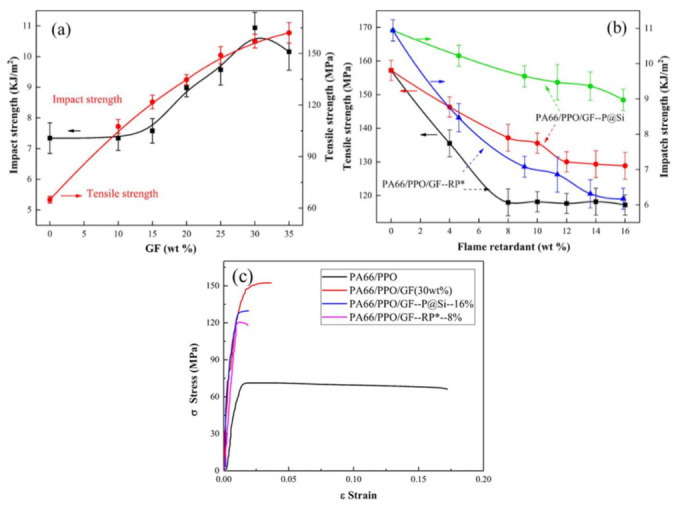
Mechanical properties of the (**a**) PA66/PPO/GF and (**b**) PA66/PPO/GF composites, and the corresponding stress-strain curves (**c**).

Glass fibers were added to the PA66/PPO composites to improve the mechanical properties of the PA66/PPO composites containing halogen-free flame retardants. The tensile and impact strength of the PA66/PPO/GF composites are shown in Figure 6a, and the red and black curves indicate tensile and impact strength, respectively. The tensile strength increased parabolically with the increasing GF content. The impact strength remained basically unchanged when the GF content was less than 15 wt% and then increased rapidly with increasing GF content. The impact strength reached the maximum value when then GF was 30%. Generally speaking, when the GF content was 30wt%, PA66/PPO/GF composites had better mechanical properties. Therefore, the GF content used in the halogen-free flame-retardant PA66/PPO/GF composites was 30%.

Figure 6b shows the tensile and impact strength of PA66/PPO/GF composites containing different ratios of halogen-free flame retardants. The black and red curves indicate the tensile strength of PA66/PPO/GF–P@Si and PA66/PPO/GF–RP*, and the blue and green curves indicate their impact strength, respectively. Similarly to the PA66/PPO composites, the tensile and impact strength of PA66/PPO/GF composites descreased with increasing flame retardants contents. Nevertheless, with the same flame-retardant content, the tensile strength of the PA66/PPO/GF composites was more than twice that of the PA66/PPO composites. For example, the tensile strength of PA66/PPO/GF–P@Si–16% and the PA66/PPO/GF–RP*–8% composites were 117.23 ± 3.01 MPa and 137.19 ± 3.95 MPa, representing improvements of 103% and 115%, respectively. In addition, the impact strength of PA66/PPO/GF composites was similar to the PA66/PPO/composites without flame retardant. Therefore, the tensile and impact strength improvement was attributed to the GF.

The strain-stress curves of PA66/PPO, PA66/PPO/GF, PA66/PPO/GF–P@Si–16%, and PA66/PPO/GF–RP*–8% composites are shown in Figure 6c. It was clearly observed that the addition of GF improved the tensile stress of PA66/PPO composites and reduced the strain behavior, which was consistent with a previously published study [33]. The tensile stress and strain of the PA66/PPO/GF–P@Si–16% and PA66/PPO/GF–RP*–8% composites were reduced due to the addition of flame retardants, compared to the PA66/PPO/GF–30 wt% composites. The strain ε values of the PA66/PPO/GF–P@Si–16% and PA66/PPO/GF–RP*–8% composites were 0.02 and 0.18, respectively. Consequently, GF improved the tensile stress, whereas it did not change the fracture mode of the PA66/PPO composites containing the and P@Si and RP* flame retardants.

**Figure 7:**
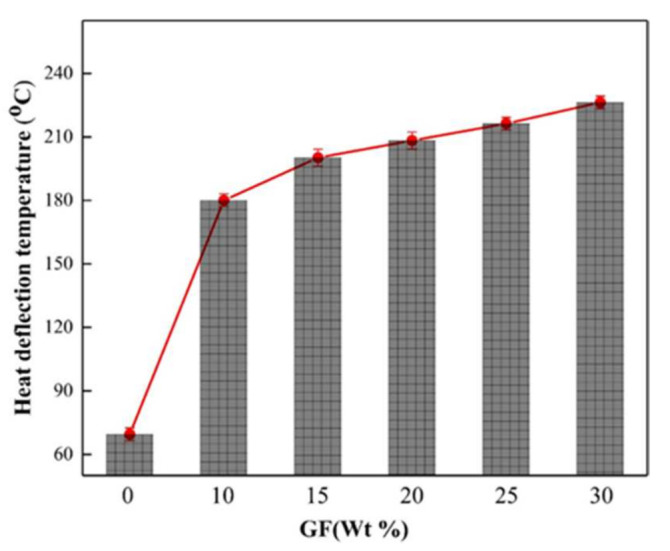
Heat deflection temperature of PA66-PPO with different GF ratios.

Figure 7 shows the thermal deformation temperature (HDT) of PA66-PPO composites with different GF ratios. The HDT of PA66 was approximate 50 °C, based on [34]. The HDT of GF-free PA66-PPO composites was approximately 72 °C and this was higher due to the higher glass transformation temperature of PPO [35]. The HDT of PA66-PPO containing GF continuously increased with increasing GF contents, meaning that the GF improved the heat properties of PA66/PPO composites. The HDT of PA66-PPO composite reached 225 °C, showing an improvement of 200%, when the GF content was 30 wt%. This phenomenon indicates that GF remarkably improved the HDT property.

**Figure 8:**
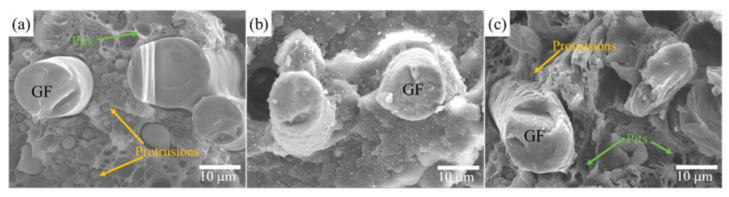
SEM images of the notch impact surfaces of the PA66/PPO/GF composites (**a**) PA66/PPO/GF, (**b**) PA66/PPO/GF–P@Si–16%, and (**c**) PA66/PPO/GF–RP*–8%.

Figure 8 shows the SEM microstructures of the notch impact surfaces of the PA66/PPO/GF composites containing the flame retardants. GF was evenly embedded in the PA66/PPO composites, which dramatically improved the comprehensive mechanical properties of the composites. In Figure 8a, many protrusions and pits were found except for the GF, which proved beneficial in improving the tensile and impact strength of the PA66/PPO composites. However, the relatively smooth surface of PA66/PPO/GF–P@Si–16% composites showed brittle fracture characteristics, as shown in Figure 8b. Therefore, the addition of P@Si flame retardant caused the fracture mode of the composite to change from a ductile to a brittle fracture mode, resulting in decreased mechanical properties. Compared to the PA66/PPO/GF–P@Si–16% composites, a few protrusions and pits were observed in the PA66/PPO/GF–RP*–8% composites, as shown in Figure 8c, implying that the PA66/PPO/GF–RP*–8% composites included partially ductile fracture modes. Although the mechanical properties of the PA66/PPO/GF–RP*–8% composites were reduced, they were better than those of the PA66/PPO/GF–P@Si–16% composites. Consequently, adding GF could improve the mechanical properties of the PA66/PPO composites but did not change the PA66/PPO composites’ fracture mode.

### 3.4. Thermal Analysis

**Figure 9:**
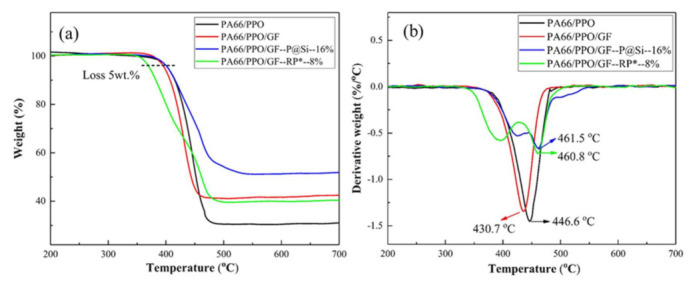
TAG (**a**) and DTG (**b**) curves of samples.

The influence of the GF, P@Si, and RP* flame retardants on the decomposition of the PA66/PPO, PA66/PPO, PA66/PPO/GF, PA66/PPO/GF–P@Si–16%, and PA66/PPO/GF–RP*–8% composites were characterized. All samples were heated in a nitrogen (N_2_) atmosphere from room temperature to 800 °C. The corresponding TAG and DTG curves of the PA66/PPO, PA66/PPO/GF, PA66/PPO/GF–P@Si–16%, and PA66/PPO/GF–RP*–8% composites are shown in Figure 9. Due to the “wick effect” of GF [36,37], the thermal stability, such as the initial decomposition temperature (T_loss_ 5 wt%), the maximum mass loss rate (R_max_), and the maximum mass loss rate temperature (Tmax), of the PA66/PPO/GF composites were all reduced compared to the PA66/PPO composites. The detailed data are listed in Table 3.

For PA66/PPO/GF–RP*–8% composites, the initial decomposition temperature was lower than that of other composites due to the decomposition of the Al(OH)_3_ coating in the RP* flame retardant. Nevertheless, the maximum decomposition temperatures of PA66/PPO/GF–P@Si–16% and the PA66/PPO/GF–RP*–8% composites were 461.5 °C and 460.8 °C, which were higher than those of the two other composites, respectively. In addition, the maximum mass loss rates of the PA66/PPO/GF–RP*–8% composites and the PA66/PPO/GF–P@Si–16% composites were lower than those of the two other composites. Therefore, the use of P@Si and the RP* could effectively improve the maximum decomposition temperatures and inhibit the weight loss of PA66/PPO/GF composites, showing an excellent flame-retardant effect.

## 4. Conclusions

PA66/PPO composites were prepared with the inclusion of P@Si and RP* flame retardants and GF, respectively. Our conclusions were as follows:

1. P@Si and RP* Flame retardants effectively improved the flame resistance of the PA66/PPO composites, and the LOI value increased linearly with the increasing contents of the P@Si and RP* flame retardants. However, the mechanical performance of the PA66/PPO composites was reduced due to the transition from a ductile fracture mode to a brittle fracture mode.
2. The addition of GF strengthened the mechanical properties of PA66/PPO composites. Although the mechanical properties of the PA66/PPO/GF composites containing P@Si and RP* flame retardants were reduced, these properties were higher than those of the PA66/PPO composites without GF. In addition, GF improved the HDT of PA66/PPO composites.
3. The maximum decomposition temperatures of PA66/PPO/GF composites were improved, and their maximum mass loss rates were reduced, indicating that the PA66/PPO/GF–P@Si–16% and PA66/PPO/GF–RP*–8% composites had excellent flame-retardant effects.

## Figures and Tables

**Table 1:** Composition of flame-retardant materials.

Materials	Abbreviation	Composition	Serial No.	Company
Polyamide 66	PA66	Density: 1.12 g/cm^3^	Industrial-Grade	Pingdingshan Shenma Engineering Plastics Co., Ltd., Pingdingshan, China
Polyphenylene Oxide	PPO	Density: 1.05 g/cm^3^	Industrial-Grade	Nantong Xingchen Synthetic Materials Co., Ltd., Nantong, China
HIPS-g-MAH compatibilizer	-	4–8 wt% MAH in HIPS-g-MAH	-	Homemade [17]
Phosphorus-Silicon	P@Si	98 wt% aluminum diethylphosphinate (ADP) + 2 wt% polydimethylsiloxane.	WR6002	Zibo Wanrong Chemical Co., Ltd., Zibo, China
Al(OH)_3_-coated red phosphorus	RP*	60 wt% Al(OH)_3_ coated 40 wt% red phosphorus.	FRM-150B	Zibo Wanrong Chemical Co., Ltd., Zibo, China
Glass fiber	GF	Moisture content < 0.05%, Sizing content 0.45 ± 0.10	568H	Jushi Group Co., Ltd., Jiaxing, China

**Table 2:** LOI values and UL-94 level of the PA66/PPO–P@Si and PA66/PPO–RP* composites.

Samples	LOI (%)	UL-94 Level
PA66/PPO	24.0 ± 0.5	V-2
PA66/PPO–P@Si–4%	25.8 ± 0.8	V-2
PA66/PPO–P@Si–8%	28.5 ± 0.6	V-1
PA66/PPO–P@Si–12%	29.3 ± 0.9	V-1
PA66/PPO–P@Si–16%	30.5 ± 0.7	V-0
PA66/PPO–P@Si–20%	33.2 ± 0.6	V-0
PA66/PPO–RP*–4%	28.1 ± 0.8	V-1
PA66/PPO–RP*–8%	34.4 ± 0.7	V-0
PA66/PPO–RP*–10%	38.5 ± 0.6	V-0
PA66/PPO–RP*–12%	39.7 ± 0.9	V-0
PA66/PPO–RP*–14%	41.0 ± 0.7	V-0

**Table 3:** Thermal decomposition data of the samples.

Samples	T_loss 5 wt%_ (°C)	T_max_ (°C)	R_max_ (%/min^−1^)
PA66/PPO	403.9	446.6	1.451
PA66/PPO/GF	397.4	430.7	1.344
PA66/PPO–P@Si–16%	403.4	461.5	0.664
PA66/PPO–RP*–8%	371.7	460.8	0.720

## Data Availability

The data presented in this study are available on request from the corresponding author.
